# Metabolic dysfunction associated steatotic liver disease in patients with plaque psoriasis: a case–control study and serological comparison

**DOI:** 10.3389/fmed.2024.1400741

**Published:** 2024-05-15

**Authors:** Zheng Lin, Yue-yi Shi, Lu-yan Yu, Chen-xi Ma, Si-yi Pan, Yuan Dou, Qiu-jun Zhou, Yi Cao

**Affiliations:** ^1^First Clinical Medical College of Zhejiang Chinese Medical University, Hangzhou, Zhejiang, China; ^2^The First Affiliated Hospital of Zhejiang Chinese Medical University, Hangzhou, Zhejiang, China

**Keywords:** plaque psoriasis, MASLD, lean MASLD, CYFRA21-1, cytokeratin-19

## Abstract

**Background:**

The relationship between plaque psoriasis and both MASLD and lean MASLD has not been sufficiently explored in the current literature.

**Method:**

This retrospective and observational study was carried out from January 2021 to January 2023 at The First Affiliated Hospital of Zhejiang Chinese Medical University. Patients diagnosed with plaque psoriasis and a control group consisting of individuals undergoing routine physical examinations were enrolled. The incidence of MASLD and lean MASLD among these groups was compared. Additionally, patients with plaque psoriasis were divided into those with MASLD, those with lean MASLD, and a control group with only psoriasis for a serological comparative analysis.

**Results:**

The incidence of MASLD in the observation group and the control group was 43.67% (69/158) and 22.15% (35/158), respectively (*p* < 0.01). Furthermore, the incidence of lean MASLD within the observation group and the control group was 10.76% (17/158) and 4.43% (7/158), respectively (*p* < 0.01). After controlling for potential confounding variables, plaque psoriasis was identified as an independent risk factor for MASLD with an odds ratio of 1.88 (95% cl: 1.10–3.21). In terms of serological comparison, compared to the simple psoriasis group, we observed a significant elevation in the tumor marker CYFRA21-1 levels in both groups compared to the control group with simple psoriasis (*p* < 0.01). Moreover, the MASLD group exhibited elevated levels of inflammatory markers and psoriasis score, whereas these effects were mitigated in the lean MASLD group.

**Conclusion:**

The prevalence of MASLD and lean MASLD is higher among patients with psoriasis. Those suffering from psoriasis along with MASLD show increased psoriasis scores and inflammatory markers compared to those without metabolic disorders. MASLD likely worsens psoriasis conditions, indicating the necessity of targeted health education for affected individuals to reduce the risk of MASLD, this education should include guidelines on exercise and diet. In serological assessments, elevated levels of cytokeratin 19 fragment (CYFRA21-1) were noted in both MASLD and lean MASLD groups, implying a potential synergistic role between psoriasis and MASLD.

## 1 Introduction

Psoriasis is a chronic skin disease mediated by the immune system, affecting roughly 0.11% of the population in East Asia (1). Population-based research across six cities reveals a psoriasis prevalence of about 0.47% in China (2). The disease imposes a significant burden on patients, including economic and psychological strains such as increased absenteeism, high treatment costs, reduced sleep quality, and social stigma (3–5).

The pathogenesis of psoriasis involves genetic predispositions, environmental triggers, immune dysregulation, and other factors (6). Research into psoriasis has progressively clarified its pathogenesis. Studies have shown that psoriasis pathogenesis is controlled by a dynamic interplay between extracellular cytokine pathways and intracellular signaling molecules (7). The key role of the tumor necrosis factor-alpha (TNF-α)/interleukin-23 (IL-23)/interleukin-17 (IL-17) axis in psoriasis, especially plaque psoriasis, is well-documented (8, 9). IL-23, produced by dendritic cells, activates intracellular signaling pathways when it binds to receptors on CD4+ helper T cells (Th17), leading to IL-17 secretion by these cells (10). IL-17 binding to keratinocyte receptors triggers the release of inflammatory mediators including TNF-α (11). TNF-α not only promotes the release of IL-23 from dendritic cells but also interacts with keratinocytes, creating an inflammatory feedback loop and promoting abnormal keratinocyte proliferation (12, 13). Plaque psoriasis, the most common variant, features pruritus, xerosis, and scaly plaques. Other forms include erythrodermic psoriasis, pustular psoriasis, and psoriatic arthritis (14).

Furthermore, the metabolic complications associated with psoriasis have gained significant attention, increasingly viewed as an immunometabolic disorder (15). Key metabolic pathways such as glycolysis, the tricarboxylic acid cycle, lipid metabolism, and amino acid metabolism are vital in regulating keratinocytes and immune cells (16, 17). The link between psoriasis and metabolic complications may stem from a common genetic basis, chronic inflammation, immune regulation, and oxidative stress (18). An expanding corpus of research strongly supports the relationship between psoriasis and metabolic disorders (19–21), making it crucial to further explore this connection to improve academic insight.

Nonalcoholic fatty liver disease (NAFLD) is characterized by triglyceride accumulation in the liver (22). The prevalence of NAFLD is rising, largely due to obesity and metabolic syndrome. Estimates suggest that up to 32% of adults worldwide are affected, highlighting significant global health concerns (23). The criteria for diagnosing NAFLD focus on liver steatosis while ruling out alcohol consumption and other hepatotoxic causes, thus providing a clear diagnosis (22). In 2023, the American, European, and Latin American Liver Societies implemented a modified Delphi process to update the terminology and definition of fatty liver disease. Consequently, NAFLD has been redefined as MASLD, which includes traditional hepatic steatosis and at least one of five common cardiometabolic risk factors (24).

Since its introduction, MASLD has shown several advantages over the traditional NAFLD terminology, including improved identification of the risk for hepatic and extrahepatic mortality, disease associations, and detection of high-risk individuals (25). The MASLD diagnostic criteria have gained broad acceptance across numerous prestigious medical societies for their precision, the simplified process by eliminating the need to exclude other liver diseases, and decreased stigma associated with the diagnosis (25). Despite the known link between MASLD and obesity, the emergence of lean MASLD has attracted significant attention (26). The criteria for diagnosing lean MASLD specify an individual with a normal Body Mass Index (BMI) diagnosed with MASLD. Previous research indicates a relationship between the onset and severity of psoriasis with metabolic syndrome and NAFLD (27, 28). This study revisits these associations using the MASLD diagnostic criteria, marking it as the first to explore the connection with psoriasis under these new guidelines. Therefore, this case–control study aims to examine the link between plaque psoriasis and both MASLD and lean MASLD, seeking to identify potential serum-specific markers in patients with plaque psoriasis affected by MASLD or lean MASLD and clarify the potential mechanisms of their interaction.

## 2 Methods

### 2.1 Selection of patients

**Figure 1:**
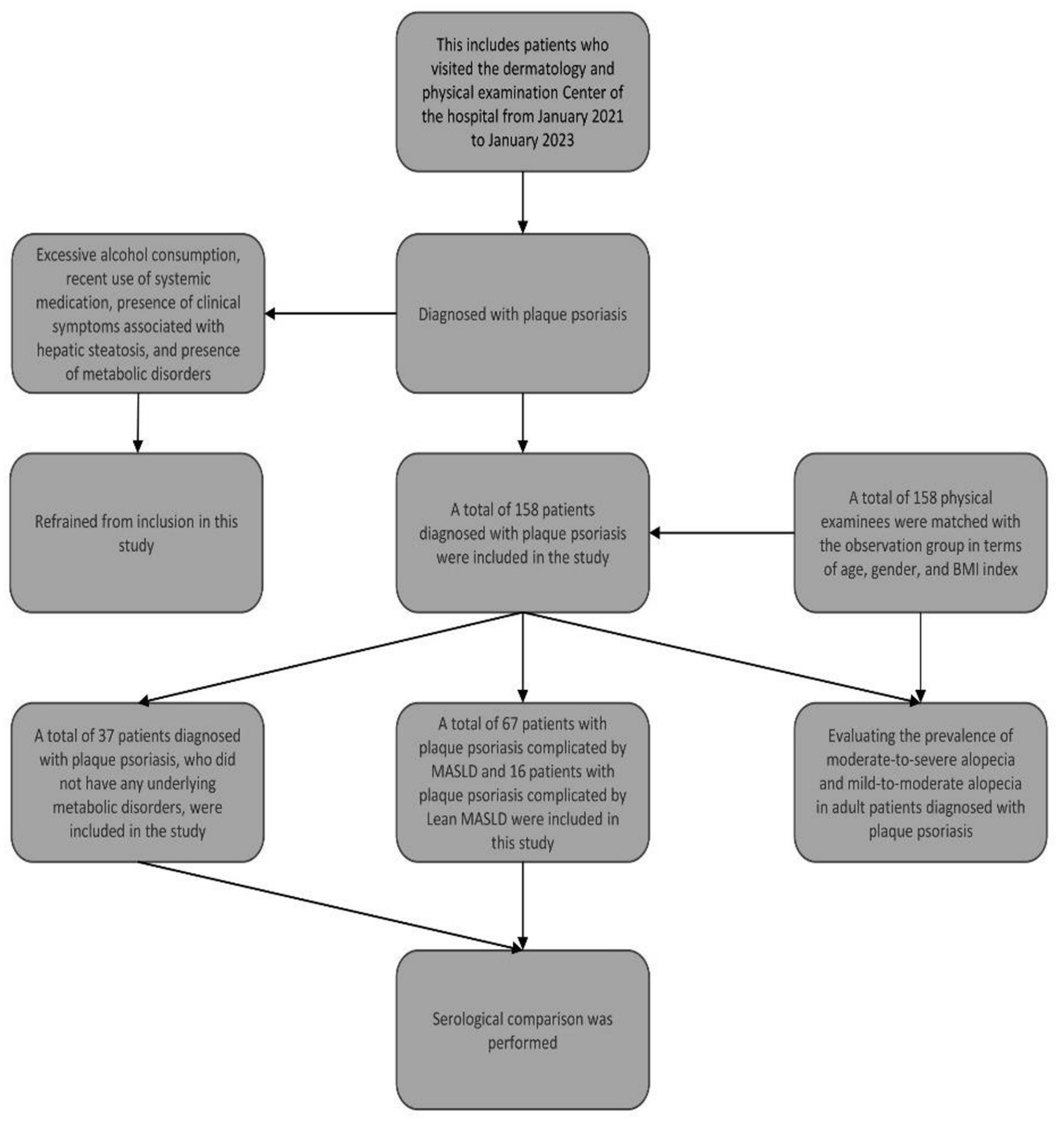
Flow chart of research methods.

We carried out a retrospective, observational study on 158 patients diagnosed with plaque psoriasis at The First Affiliated Hospital of Zhejiang Chinese Medical University, China, from January 2021 to January 2023, referred to as the observation group. An equal number of individuals undergoing routine physical examinations at the Physical Examination Center of The First Affiliated Hospital of Zhejiang Chinese Medical University, China, formed the control group. The inclusion criteria were as follows: individuals aged over 18 years with a clinical diagnosis of plaque psoriasis; control group subjects matched with the observation group on age, gender, and BMI. The exclusion criteria included: individuals with significant alcohol consumption (more than 20 g/day) and other clinical conditions like viral hepatitis that could induce hepatic steatosis, known risk factors for steatotic liver disease (SLD) (29); those on systemic therapies such as methotrexate, cyclosporine, or biologics within the last month, which could confound liver disease or psoriatic symptoms (30); and those with conditions like hypothyroidism, tumors, or type 1 diabetes, which are associated with metabolic diseases (31); as well as pregnant, expecting, or lactating women, and individuals with severe infections, tuberculosis, or skin cancers. From the total of 158 patients, we recruited 37 with plaque psoriasis without metabolic disease (simple psoriasis group), 67 with plaque psoriasis and MASLD (MASLD group), and 16 with plaque psoriasis and lean MASLD (lean MASLD group) for serological analysis. Patients with incomplete data were excluded. The study protocol received approval from the Institutional Review Board, and the ethics committee waived the requirement for informed consent from participants, given the retrospective analysis of an existing database (Figure 1).

### 2.2 Research methodology

Clinical and laboratory parameters were recorded for all participants, including age, sex, weight, height, BMI, and medical history of hypertension, diabetes, and hyperlipidemia. Additionally, we recorded results from liver ultrasonography for SLD diagnosis, systolic and diastolic blood pressure, fasting blood glucose, blood triglycerides, total cholesterol, high-density lipoprotein cholesterol (HDL-C), and low-density lipoprotein cholesterol (LDL-C). We also calculated the incidence of MASLD and lean MASLD.

In parallel, laboratory serum data was collected for serological analysis. This included white blood cell count, absolute counts of neutrophils, lymphocytes, monocytes, eosinophils, and basophils, and hypersensitive C-reactive protein. Tumor markers such as alpha fetoprotein (AFP), carbohydrate antigen 19 (CA19-9), carbohydrate antigen 242 (CA242), carbohydrate antigen 50 (CA50), carbohydrate antigen 72-4 (CA72-4), CYFRA21-1 protein marker (CYFRA21-1); nerve-specific enolase marker (NSE) and squamous cell carcinoma-related antigen (SCC-Ag) were also evaluated. Biochemical markers measured comprised blood glucose levels, triglyceride levels, total cholesterol levels; high-density lipoprotein cholesterol levels (HDL-C); low-density lipoprotein cholesterol levels (LDL-C); total bilirubin, direct bilirubin, indirect bilirubin, glutamic oxalacetic transaminase (GOT), alanine aminotransferase (ALT), aspartate aminotransferase (AST), and AST: ALT ratios. Body surface area (BSA) and psoriasis area and severity index (PASI) were used to assess the severity of skin lesions in patients with plaque psoriasis. We collected these markers because routine blood tests cover most inflammatory markers, while biochemical routines encompass most metabolic factor markers. TNF-α exhibits diverse immunomodulatory effects and possesses the ability to recognize and eliminate tumor cells. Considering the key role of TNF-α in psoriasis pathogenesis (8) and its function in TNF-α-mediated apoptosis in MASLD (32), Previous studies have also elucidated the correlation between tumor biomarkers and psoriasis, such as squamous cell carcinoma-related antigens and psoriasis (33). Therefore, we hypothesized that psoriasis and MASLD could alter tumor marker profiles, prompting an investigation into their potential linkage. All serological parameters were collected after an overnight fast of at least 8 h from venous blood samples drawn from the antecubital vein, analyzed using standard procedures at our central laboratory.

The diagnosis of psoriasis is made by experienced dermatologists based on the 2023 edition of the guideline for the diagnosis and treatment of psoriasis in China (34), this process includes evaluating the characteristics of skin lesions and using auxiliary dermoscopy. The diagnosis of SLD is determined by experienced radiologists through ultrasound examination, following the guidelines for assessing and managing non-alcoholic fatty liver disease in the Asia-Pacific region (35). The diagnosis of MASLD and related metabolic diseases relies on results from the latest Delphi procedure (24). However, for classifying overweight, we use the obesity standard released by the Chinese Nutrition Society in June 2022, which better reflects the anthropometric characteristics of Chinese individuals (36) (Refer to Tables 1, 2 for diagnostic criteria).

**Table 1:** Diagnostic criteria for psoriasis and MASLD-related diseases by Chinese Society of Dermatology (34) and Asia-Pacific Working Party on NAFLD (35) and MASLD nomenclature consensus group (24).

Illness	Diagnostic criteria
Plaque psoriasis	Dark red lesions exhibiting infiltrative erythema, overlaid with white or silver-white scales and accompanied by wax droplets, film phenomenon, and punctate hemorrhage. Dermoscopy: Psoriasis vulgaris exhibits a characteristic dermoscopic pattern of evenly distributed punctate and spherical blood vessels on a red background, accompanied by diffuse white scales. Under magnification exceeding 50 times, these punctate and spherical blood vessels manifest as clusters of capillaries or glomerular vessels.
Obesity	BMI ≥ 28 kg/m^2^
SLD	Ultrasonic diagnostic criteria: Differential echoes of the liver and kidney were observed, with an increased brightness in liver echo; the intrahepatic duct structure appeared unclear; and there was attenuation of the far-field echo in the liver.
MASLD	SLD and more than one of five Cardiometabolic criteria
Lean MASLD	SLD and more than one of five Cardiometabolic criteria and BMI ≤ 24 kg/m^2^
Cryptogenic SLD	SLD without Cardiometabolic criteria
Metabolic syndrome	More than three of five Cardiometabolic criteria

BMI, body mass index; SLD, steatotic liver disease; MASLD, metabolic dysfunction-associated steatotic liver disease.

**Table 2:** Adult criteria for five Cardiometabolic criteria by MASLD nomenclature consensus group (24, 36).

Cardiometabolic criteria	Adult criteria
Obesity	BMI ≥ 24 kg/m^2^
Hypertension	Blood pressure ≥ 130/85 mmHg OR specific antihypertensive drug treatment
Diabetes	Fasting serum glucose ≥ 5.6 mmol/L [100 mg/dL] OR type 2 diabetes OR treatment for type 2 diabetes
High triglycerides	Plasma triglycerides ≥ 1.70 mmol/L [150 mg/dL] OR lipid lowering treatment
Low high-density lipoprotein cholesterol	Plasma HDL-cholesterol ≤ 1.0 mmol/L [40 mg/dL] (Male) and ≤ 1.3 mmol/L [50 mg/dL] (Female) OR lipid lowering treatment

BMI, body mass index; HbA1C, Hemoglobin A1C.

### 2.3 Methodology for statistical analysis

Continuous data were presented as mean ± standard deviation, and categorical data were expressed as the number of patients (percentage). The normality of continuous data was verified using the Shapiro test and histograms, with *p* > 0.05 indicating a normal distribution. Differences between groups were analyzed using the t-test for normally distributed data, the Wilcoxon Mann–Whitney test for skewed data, and the Pearson chi-square test for nonparametric data; statistical significance was defined as *p* < 0.05. Logistic regression was utilized to explore the independent relationship between plaque psoriasis and MASLD, with MASLD as the dependent variable, adjusting for factors such as age, hypertension, diabetes, overweight status, low HDL-C, and high triglycerides. Data analysis was performed using SPSS 25 (SPSS Inc., Chicago, IL) and R version 4.3.1 (R Foundation for Statistical Computing).

## 3 Results

### 3.1 Case control study

**Figure 2:**
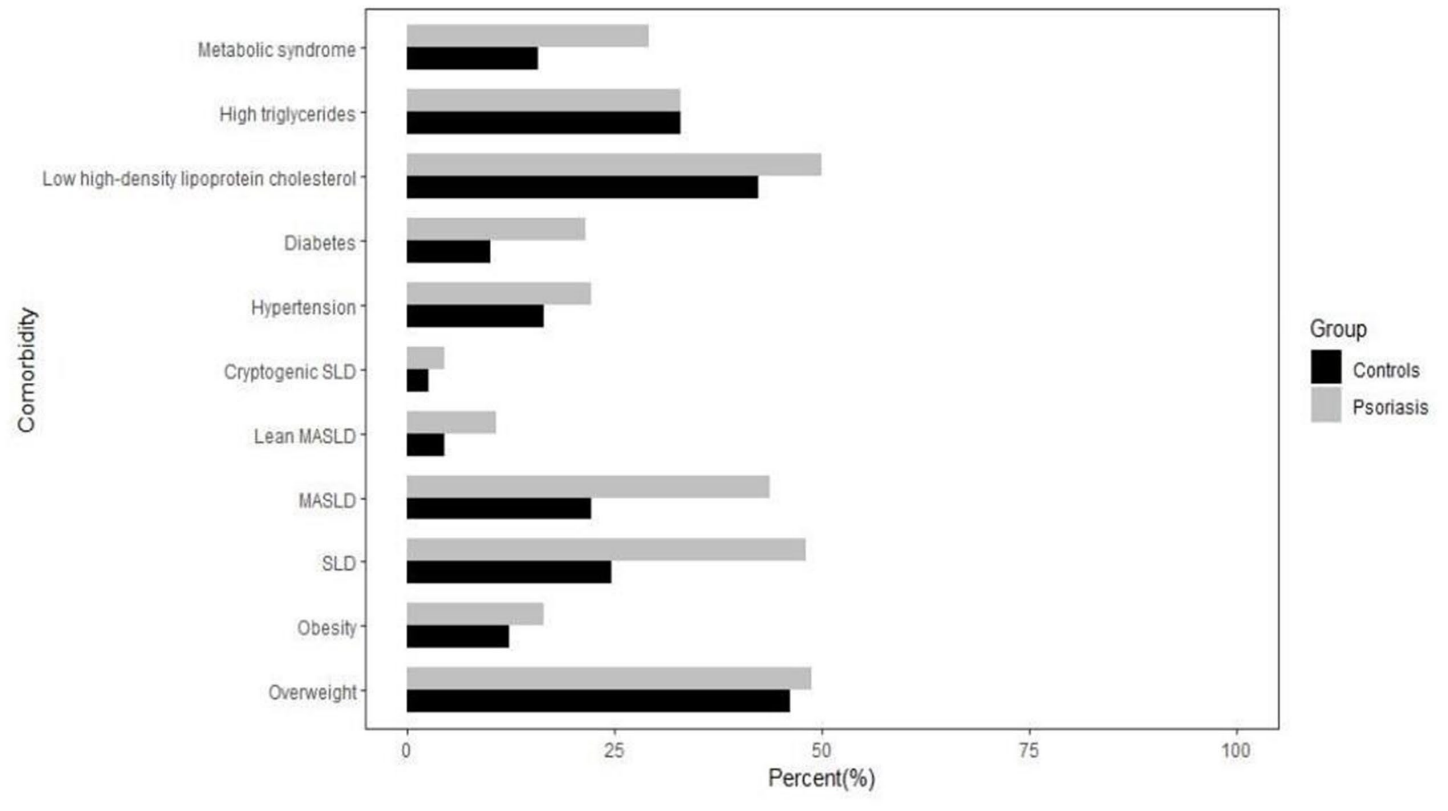
The main clinical characteristics of the study population.

This study enrolled 158 patients with plaque psoriasis (observation group) and 158 gender-, age-, and BMI-matched healthy controls (control group) who visited The First Affiliated Hospital of Zhejiang Chinese Medical University between January 2021 and January 2023. The baseline characteristics of the study participants are presented in Table 3, while the main clinical features are presented in Table 4 and visualized through a histogram (Figure 2).

**Table 3:** Relevant baseline characteristics of the study cohort.

	Psoriasis (Group A) (*N* = 158)	Controls (Group B) (*N* = 158)	*p*-value
Age at enrollment (years)	47.10 ± 13.66	47.66 ± 14.75	0.64
Height (cm)	167.92 ± 7.51	167.02 ± 7.82	0.23
Weight (kg)	69.91 ± 14.59	67.48 ± 12.54	0.16
BMI (kg/m^2^)	24.68 ± 4.25	24.09 ± 3.54	0.20
Sex/gender, Male, *n* (%)	107 (67.72)	96 (60.76)	0.20

Data are shown as mean ± standard deviation or *n* (%); AST, aspartate aminotransferase; ALT, alanine aminotransferase; BMI, body mass index.

**Table 4:** The main clinical characteristics of the study population.

	Psoriasis (Group A) (*N* = 158)	Controls (Group B) (*N* = 158)	*p*-value
Overweight, *n* (%)	77 (48.73)	73 (46.20)	0.65
Obesity, *n* (%)	26 (16.46)	19 (12.30)	0.26
SLD, *n* (%)	76 (48.10)	39 (24.68)	<0.01
MASLD, *n* (%)	69 (43.67)	35 (22.15)	<0.01
Lean MASLD, *n* (%)	17 (10.76)	7 (4.43)	<0.05
Cryptogenic SLD, *n* (%)	7 (4.43)	4 (2.53)	0.36
Hypertension, *n* (%)	35 (22.15)	26 (16.46)	0.20
Diabetes, *n* (%)	34 (21.52)	16 (10.13)	<0.01
Low high-density lipoprotein cholesterol, *n* (%)	79 (50.00)	67 (42.41)	0.18
High triglycerides, *n* (%)	52 (32.91)	52 (32.91)	1.00
Metabolic syndrome, *n* (%)	46 (29.11)	25 (15.82)	<0.01

Data are shown as *n* (%); CI, confidence interval; OR, odds ratio; SLD, steatotic liver disease; MASLD, metabolic dysfunction-associated steatotic liver disease.

The age, height, weight, gender, and BMI did not exhibit any statistically significant differences between the two groups (*p* > 0.05), indicating that the baseline characteristics of the observation group and control group were comparable and well-matched.

The incidence of MASLD in the observation group (43.67%, 69/158) was significantly higher than that in the control group (22.15%, 35/158) (*p* < 0.01). The occurrence of lean MASLD in the observation group (10.76%, 17/158) was significantly higher compared to the control group (4.43%, 7/158) (*p* < 0.05). Furthermore, compared to the control group, there was a significant increase in both diabetes and metabolic syndrome incidences within the observation group (*p* < 0 0.05). However, no significant differences were found between groups concerning overweight, obesity, hypertension, low high-density lipoprotein cholesterol levels or hypertriglyceridemia incidences (*p* > 0 0.05).

**Figure 3:**
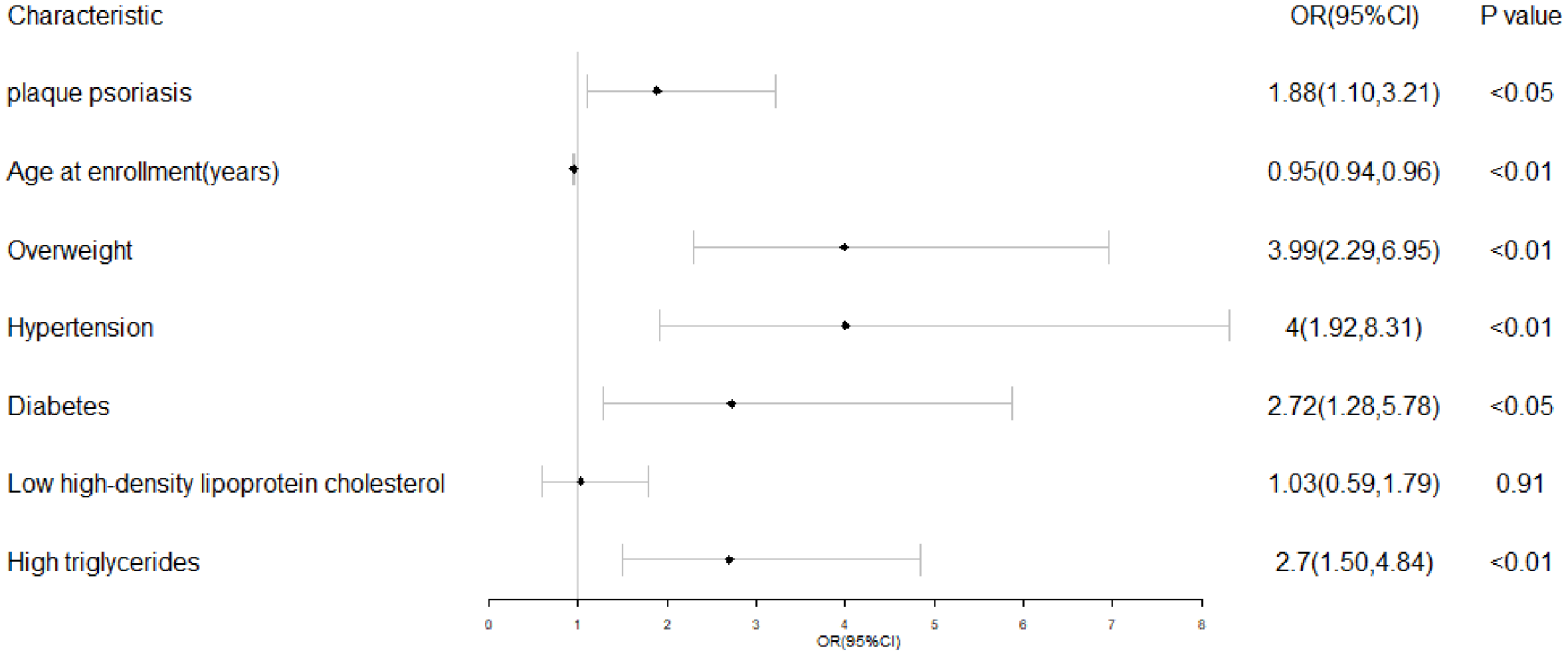
The results of multivariate logistic regression analysis.

After adjusting for age, hypertension, diabetes, overweight, low HDL-C, and hypertriglyceridemia, logistic regression analysis was employed to examine the independent association between plaque psoriasis and MASLD (with MASLD as the dependent variable). The results are presented in Table 5 and visually represented using a forest plot (Figure 3).

**Table 5:** Independent predictors of MASLD.

Group A and B (*n* = 316)	*p*-value	OR	95%CL
plaque psoriasis (yes or no)	<0.05	1.88	1.10–3.21
Age at enrollment (years)	<0.01	0.95	0.94–0.96
Overweight (yes or no)	<0.01	3.99	2.29–6.95
Hypertension (yes or no)	<0.01	4.00	1.92–8.31
Diabetes (yes or no)	<0.05	2.72	1.28–5.78
Low high-density lipoprotein cholesterol (yes or no)	0.91	1.03	0.59–1.79
High triglycerides (yes or no)	<0.01	2.70	1.50–4.84

Sample size, *n* = 316. Data are expressed as odds ratios and 95% confidence intervals (CI) calculated via logistic regression analysis. The dependent variable of this regression model was the presence of MASLD.

The association between plaque psoriasis and MASLD was examined in Table 5 (OR = 1.88, 95%CL: 1.10–3.21), after adjusting for potential confounders. Logistic regression analysis revealed significant associations of plaque psoriasis, overweight, hypertension, diabetes, and abnormal triglycerides with MASLD; however, no association was observed with abnormal HDL cholesterol.

### 3.2 Serological comparison

Serological comparisons were conducted among three subgroups: 37 patients with plaque psoriasis without metabolic disease (simple psoriasis group), 67 patients with plaque psoriasis accompanied by metabolic-associated fatty liver disease (MASLD group), and 16 patients with plaque psoriasis accompanied by lean MASLD group.

As presented in Table 6, Simultaneously, we observed a significant elevation in the tumor marker CYFRA21-1 levels in both groups compared to the control group with simple psoriasis (*p* < 0.01). Given the elevated levels of CYFRA21-1 observed in both MASLD and lean MASLD, we performed a correlation analysis between CYFRA21-1 and PASI, BSA, C-reactive protein (hs-CRP), and disease duration. However, the results indicated only weak positive correlations (cor < 0.3) with these indicators; hence they were not individually included in the table.

**Table 6:** Tumor index of psoriasis with no comorbidities group and psoriasis with MASLD group and lean MASLD group.

	Simple psoriasis (Group C) (*N* = 37)	MASLD (Group D) (*N* = 67)	lean MASLD (Group E) (*N* = 16)	*p*-value for C and D	*p*-value for C and E
Alpha-fetoprotein (ng/mL)	2.52 ± 0.67	3.04 ± 1.26	2.68 ± 0.97	<0.05	0.75
CA19-9 (U/mL)	7.17 ± 6.4	9.09 ± 9.39	10.43 ± 10.24	0.48	0.37
CA242 (U/mL)	5.12 ± 2.57	4.86 ± 3.54	5.95 ± 3.65	0.29	0.35
CA50 (U/mL)	5.62 ± 3.48	6.63 ± 4.93	6.98 ± 4.45	0.36	0.32
CA72-4 (U/mL)	3.67 ± 13.26	3.67 ± 6.06	3.08 ± 6.32	0.42	0.26
CYFRA21-1 (ng/mL)	1.27 ± 0.79	1.6 ± 0.82	1.68 ± 0.75	<0.01	<0.01
Neuro-specific enolase (ng/mL)	3.78 ± 2.64	4 ± 2.54	4.03 ± 1.91	0.29	0.20
SCC-Ag (ng/mL)	7.26 ± 13.64	5.71 ± 9.99	8.78 ± 15.35	0.47	0.32

CA19-9, CA19-9 antigen; CA242, CA242 antigen; CA50, CA50 antigen; CA72-4, CA72-4 antigen; CYFRA21-1,CYFRA21-1 protein marke; SCC-Ag, squamous cell carcinoma-related antigens.

As presented in Tables 7–9, In terms of serological comparison, compared to the simple psoriasis group, the MASLD group exhibited higher PASI (*p* < 0.05) and BSA (*p* < 0.01); elevated white blood cell count (*p* < 0.05), neutrophil count (*p* < 0.01); increased high-sensitivity C-reactive protein levels (*p* < 0.05); blood glucose (*p* < 0.01), triglyceride (*p* < 0.01), total cholesterol (*p* < 0.01), and low-density lipoprotein cholesterol levels (*p* < 0.01) were also elevate, while AST: ALT ratio was lower (*p* < 0.01). There were no statistically significant differences observed in the remaining indicators (*p* > 0.05).

**Table 7:** Baseline data and years of onset and psoriasis score of psoriasis with no comorbidities group and psoriasis with MASLD group and lean MASLD group.

	Simple psoriasis (Group C) (*N* = 37)	MASLD (Group D) (*N* = 67)	Lean MASLD (Group E) (*N* = 16)	*p*-value for C and D	*p*-value for C and E
Age at enrollment (years)	42.16 ± 14.47	47.54 ± 11.78	50.88 ± 12.26	<0.05	<0.05
Height (cm)	164.84 ± 6.85	169.07 ± 7.49	165.44 ± 7.84	<0.01	0.78
Weight (kg)	61.76 ± 11.19	76.46 ± 13.22	62.91 ± 6.11	<0.01	0.70
BMI (kg/m^2^)	22.65 ± 3.32	26.66 ± 3.7	22.95 ± 0.82	<0.01	0.72
Years of onset (years)	9.46 ± 10.22	11.39 ± 10.76	11.69 ± 13.82	0.15	0.62
BSA	29.41 ± 13.4	38.98 ± 17.71	39.56 ± 15.06	<0.01	<0.05
PASI	17.46 ± 8.77	22.14 ± 9.63	20.39 ± 7.97	<0.05	0.26

BSA, body surface area; PASI, psoriasis area and severity index.

**Table 8:** Blood routine examination of psoriasis with no comorbidities group and psoriasis with MASLD group and lean MASLD group.

	Simple psoriasis (Group C) (*N* = 37)	MASLD (Group D) (*N* = 67)	Lean MASLD (Group E) (*N* = 16)	*p*-value for C and D	*p*-value for C and E
White blood cell count (*10^9^/L)	5.81 ± 1.61	6.85 ± 1.87	6.66 ± 2.03	<0.05	0.11
Neutrophil count (*10^9^/L)	3.46 ± 1.16	4.31 ± 1.54	4.22 ± 1.97	<0.01	0.25
Lymphocyte count (*10^9^/L)	1.67 ± 0.65	1.83 ± 0.72	1.75 ± 0.58	0.20	0.51
Monocyte count (*10^9^/L)	0.48 ± 0.18	0.5 ± 0.15	0.47 ± 0.13	0.24	0.73
Eosinophilic counts (*10^9^/L)	0.17 ± 0.15	0.17 ± 0.12	0.19 ± 0.17	0.60	0.88
Basophil count (*10^9^/L)	0.03 ± 0.02	0.04 ± 0.06	0.03 ± 0.02	0.56	0.86
High sensitivity C-reactive protein (mg/L)	5.21 ± 19.56	6.93 ± 21.19	15.82 ± 42.21	<0.05	0.06

**Table 9:** Biochemical index psoriasis with no comorbidities group and psoriasis with MASLD group and lean MASLD group.

	Simple psoriasis (Group C) (*N* = 37)	MASLD (Group D) (*N* = 67)	lean MASLD (Group E) (*N* = 16)	*p*-value for C and D	*p*-value for C and E
Blood Sugar (mmol/L)	4.8 ± 0.83	5.66 ± 1.72	5.64 ± 2.35	<0.01	0.23
Triglyceride (mmol/L)	1.29 ± 0.85	1.99 ± 1.3	1.55 ± 0.43	<0.01	<0.05
Total cholesterol (mmol/L)	4.26 ± 0.72	4.78 ± 1.09	4.66 ± 0.72	<0.01	0.16
High density lipoprotein (mmol/L)	1.17 ± 0.33	1.08 ± 0.26	1.13 ± 0.22	0.19	1.00
Low-density lipoprotein (mmol/L)	2.33 ± 0.59	2.78 ± 0.73	2.7 ± 0.53	<0.01	0.05
Total bilirubin (umol/L)	12.55 ± 5.61	12.87 ± 4.75	12.42 ± 3.84	0.63	0.93
Direct bilirubin (umol/L)	4.75 ± 2.1	5.01 ± 1.77	5.28 ± 1.48	0.42	0.37
Indirect bilirubin (umol/L)	7.8 ± 3.81	7.86 ± 3.48	7.14 ± 2.82	0.94	0.54
AST (U/L)	17.81 ± 6.79	24.54 ± 26.91	29.94 ± 50.69	0.07	0.64
ALT (U/L)	16.92 ± 13.96	35.93 ± 52.6	43.75 ± 99.48	<0.01	<0.05
AST:ALT ratio	1.29 ± 0.43	0.84 ± 0.33	0.97 ± 0.38	<0.01	<0.05

AST, aspartate aminotransferase; ALT, alanine aminotransferase.

Compared to the simple psoriasis group, the lean MASLD group exhibited a significantly higher BSA score (*p* < 0.05), while no statistically significant difference was observed in PASI (*p* = 0.26) and high-sensitivity C-reactive protein levels (*p* = 0.06). Moreover, elevated platelet count were detected in blood routine analysis (*p* < 0.05). Additionally, biochemical indexes indicated raised triglyceride levels and ALT activity (*p* < 0.05), along with a decreased AST: ALT ratio (*p* < 0.05). There were no statistically significant differences observed in the remaining indicators (*p* > 0.05).

## 4 Discussion

### 4.1 Discussion on case-control studies, focusing on lean MASLD

To our knowledge, this is the first study to apply the MASLD diagnostic criteria to investigate its correlation with psoriasis following the recent update of MASLD’s definition. Similar to earlier studies linking psoriasis with NAFLD, 48.1% of our patients with plaque psoriasis were diagnosed with SLD (37), which can be attributed to our study’s rigorous exclusion of confounding factors such as alcohol intake and certain medications. Immunosuppressives like methotrexate and cyclosporine, often used in systemic psoriasis treatment, are known to significantly contribute to liver fibrosis (30, 38). To avoid these confounding factors, we excluded participants who had used these medications in the month prior to enrollment. Patients were treated exclusively with topical agents and light therapy before entering the study, treatments which are generally considered to have minimal effects on hepatic function and metabolism (39). Assessments were conducted promptly upon admission, and since psoriasis treatment methods in this study showed limited impact on MASLD and other complications, their detailed discussion was not deemed crucial to the study’s scope. However, the potential hepatotoxicity of psoriasis medications highlights the importance of this investigation.

A novel finding from our study is the observed prevalence of MASLD among patients with plaque psoriasis at 43.67%, significantly higher than the 22.15% observed in the control group, aligning with previously reported MASLD prevalence (40). Our adjusted regression analysis further confirms a strong association between plaque psoriasis and MASLD prevalence. Notably, the prevalence of lean MASLD among psoriasis patients was significantly higher (10.76%) compared to that in healthy subjects (4.33%).

Several previous studies have consistently shown a higher prevalence of NAFLD among patients with psoriasis (41). Employing the new definition of MASLD, our findings are similar, confirming a strong association between MASLD and plaque psoriasis and highlighting the superior diagnostic coverage and accuracy of MASLD compared to NAFLD.

The link between MASLD and psoriasis could be due to the important role of insulin resistance in the metabolism of psoriatic and fatty liver conditions (42, 43), and the dysfunction in psoriasis-associated immune cells, such as dendritic cells, which stems from abnormal lipid metabolism (44, 45). Additionally, liver cell damage and the resulting cytokine secretion (e.g., FGF-7) may worsen psoriatic inflammation (46, 47), while various prominent cytokines (including IL-17) might impact metabolism (48).

Research has indicated differences in susceptibility genes and pathogenetic mechanisms between lean MASLD and MASLD (26). Our data indicate that the prevalence of lean MASLD is also elevated in patients with plaque psoriasis, yet the specific overlaps and distinctions in the comorbidity mechanisms between lean MASLD and psoriasis, and those between MASLD and psoriasis, remain to be fully understood. Our findings could guide future research in this area.

### 4.2 Discussion of serologic comparisons, focusing on CYFRA21-1, and cytokeratin-19 (CK-19)

In our study, serological comparisons were made among groups with simple psoriasis, MASLD, and lean MASLD. We found that the MASLD group had a higher BMI than the simple psoriasis group, highlighting a strong link between overweight status and MASLD. Abnormal lipid metabolism and altered liver function indicators were consistent with the expected characteristics of MASLD. Importantly, novel observations indicated that the MASLD group had increased BSA and PASI, as well as elevated inflammatory markers such as hs-CRP, compared to the psoriasis vulgaris group. These findings suggest that MASLD exacerbates skin manifestations and inflammation of psoriatic lesions. Additionally, a Mendelian randomization study suggested that a rise in white blood cell count and neutrophils could be risk factors for the onset of psoriasis (49). Thus, the increased levels of these cells observed in the MASLD group might indicate an exacerbation of psoriasis when associated with MASLD.

We also examined serologic differences between the lean MASLD group and the simple psoriasis group, taking care to exclude potential confounding factors linked to overweight in the lean MASLD group. In the lean MASLD group, higher BSA scores were noted compared to the psoriasis-alone group; however, there were no significant differences in PASI and inflammatory markers such as C-reactive protein. These findings suggest that lean-type MASLD might cause less synergistic damage to plaque psoriasis than typical MASLD, pointing to weight management as a potential comprehensive strategy for treating patients with both plaque psoriasis and MASLD. A bicenter retrospective study published in 2023 also supports our perspective on the importance of weight management in psoriasis patients (50).

Surprisingly, our study identified an increase in CYFRA21-1 levels, a tumor marker typically associated with diagnostic value in lung non-small cell carcinoma (51). This elevation was noted when comparing serological differences between MASLD, lean MASLD, and psoriasis alone. Although CYFRA21-1 showed a weak correlation with skin lesion severity (PASI and BSA) and inflammation (hs-CRP) in chronic plaque psoriasis with MASLD, without reaching abnormal values, its significance for diagnosis and prognosis may be limited. However, this increase suggests a potential link between the mechanisms of MASLD and plaque psoriasis interaction. CYFRA21-1, the soluble fragment of CK-19 found in the bloodstream (52), could indicate increased tissue apoptosis, possibly due to increased TNF-α-mediated apoptosis in patients with psoriasis (53). This process may involve abnormal activity in keratinocytes (53) and hepatic stellate cells (54), potentially contributing to the coexistence of MASLD and psoriasis. It might also reflect a rise in intracellular CK-19 content. CK-19 is prevalent in epithelium-rich tissues such as skin and liver (55, 56). Prior studies have shown that cytokeratin-6, cytokeratin-16, and cytokeratin-17, within the same family as CK-19, are upregulated in psoriasis, leading to excessive keratinocyte proliferation – supporting the involvement of the keratin system in this condition (57). Additionally, TGM2-dependent covalent CXCL12-Keratine 19 heterodimers can coat cancer cell surfaces, potentially interfering with T-cell-mediated immune responses, suggesting a role for CK-19 in immune regulation (58). Further, research has shown that the suppression of CK-19 gene expression prolongs the cell cycle in breast cancer cells, indicating that CK-19 is highly expressed in rapidly proliferating cells such as hepatocytes and keratin-forming cells (59). Confirmation of these findings would imply that targeting CK-19 expression to inhibit cell proliferation could be a promising therapeutic strategy for psoriasis and its related conditions. These findings align with a cellular experiment on keratin-17, corroborating our understanding of keratin properties and functions (60). Although these observations suggest a potential involvement of CK-19 in immune processes and cell proliferation/apoptosis related to psoriasis and MASLD, the pathogenesis of CK-19 in these conditions is yet to be thoroughly reported. Therefore, our conclusions require further validation through comprehensive cohort studies or biological experiments.

### 4.3 Summary

Our study has several limitations that merit consideration. Firstly, the study was conducted at a tertiary care center, where the majority of admitted patients presented with moderate to severe psoriasis, which may limit the generalizability of our findings. Secondly, the case–control design demonstrates a significant association but does not establish causality. Thirdly, while liver ultrasonography is sensitive and specific for diagnosing SLD, it is important to note that liver biopsy remains the gold standard for such diagnosis (35). Our study did not employ liver biopsy to assess liver fibrosis in psoriasis patients may affect the comprehensiveness of our findings. Simple steatosis is benign, but liver fibrosis is critical for the progression and prognosis of liver disease and the risk of hepatocellular carcinoma (61). We aim to address these limitations in future studies to increase the scope and depth of our research. Fourthly, the conclusions require further validation due to the small sample size of the lean MASLD group.

Despite these limitations, our study has notable strengths. It employs the novel diagnostic criteria for MASLD to explore its correlation with plaque psoriasis, representing a pioneering effort in this field as per our literature review. We specifically focused on the relationship between lean MASLD and psoriasis, laying groundwork for future investigations. Additionally, our study highlights CYFRA21-1 and CK-19 as potential markers that may help reveal the co-pathogenesis of MASLD and psoriasis. Lastly, the robust integrity of our comprehensive database, where all patients underwent detailed examinations (including liver ultrasonography) and blood samples were consistently collected at a single center.

In conclusion, our findings highlight the high prevalence of MASLD and lean MASLD among patients with plaque psoriasis. Consequently, this study advocates that treatment for patients with plaque psoriasis should extend beyond merely addressing skin lesions. It is crucial to include ultrasound screening and health education focusing on weight management. Additionally, when prescribing systemic therapy drugs such as methotrexate and cyclosporine, it is important to consider their impact on liver function. A careful assessment must be made regarding the suitability of these drugs for patients with psoriasis due to their potential liver burden. For patients with plaque psoriasis complicated by MASLD, biologics may be a preferable option, as current literature does not associate these with hepatic dysfunction. Collaboration between dermatologists and hepatologists could promote the evaluation of synergistic treatment options that benefit both conditions, ultimately improving patient outcomes. Moreover, this study opens up new avenues for research, such as exploring the potential overlap in pathogenesis between lean MASLD and psoriasis, and investigating the role of CK-19 in the development of both psoriasis and MASLD. Rigorous cohort studies and biological experiments are necessary to delve deeper into these topics.

## Data availability statement

The original contributions presented in the study are included in the article/Supplementary material, further inquiries can be directed to the corresponding author.

## Ethics statement

The studies involving humans were approved by The First Affiliated Hospital of Zhejiang Chinese Medical University ethics review board (2024-KLS-139-01). The studies were conducted in accordance with the local legislation and institutional requirements. The ethics committee/institutional review board waived the requirement of written informed consent for participation from the participants or the participants’ legal guardians/next of kin because we conducted a retrospective analysis using an existing database containing relevant data.

## Author contributions

ZL: Writing – review & editing, Writing – original draft, Software, Methodology, Formal analysis, Data curation, Conceptualization. Y-yS: Writing – review & editing, Validation, Data curation, Conceptualization. L-yY: Writing – review & editing, Investigation, Data curation. C-xM: Writing – review & editing, Investigation, Data curation. S-yP: Writing – review & editing, Validation, Conceptualization. YD: Writing – review & editing, Investigation, Data curation. Q-jZ: Writing – review & editing, Validation. YC: Writing – review & editing, Supervision, Resources, Project administration.
